# The Ability of Secretory Leukocyte Protease Inhibitor to Inhibit Apoptosis in Monocytes Is Independent of Its Antiprotease Activity

**DOI:** 10.1155/2015/507315

**Published:** 2015-07-12

**Authors:** Niamh McGarry, Catherine M. Greene, Noel G. McElvaney, Sinéad Weldon, Clifford C. Taggart

**Affiliations:** ^1^Respiratory Research Division, Department of Medicine, Education and Research Centre, Royal College of Surgeons in Ireland, Dublin 9, Ireland; ^2^Centre for Infection and Immunity, School of Medicine, Dentistry and Biomedical Sciences, Health Sciences Building, Queen's University Belfast, 97 Lisburn Road, Belfast BT9 7AE, UK

## Abstract

Secretory Leukocyte Protease Inhibitor (SLPI) is a serine protease inhibitor produced by epithelial and myeloid cells with anti-inflammatory properties. Research has shown that SLPI exerts its anti-inflammatory activity by directly binding to NF-*κ*B DNA binding sites and, in so doing, prevents binding and subsequent transcription of proinflammatory gene expression. In the current study, we demonstrate that SLPI can inhibit TNF-*α*-induced apoptosis in U937 cells and peripheral blood monocytes. Specifically, SLPI inhibits TNF-*α*-induced caspase-3 activation and DNA degradation associated with apoptosis. We go on to show that this ability of SLPI to inhibit apoptosis is not dependent on its antiprotease activity as antiprotease deficient variants of SLPI can also inhibit TNF-*α*-induced apoptosis. This reduction in monocyte apoptosis may preserve monocyte function during inflammation resolution and promote infection clearance at mucosal sites.

## 1. Introduction

Secretory Leukocyte Protease Inhibitor (SLPI) is a small molecular weight protein with multifunctional properties including antiprotease, antibacterial, antiviral, and anti-inflammatory effects [1]. The mature SLPI protein is 11.7 kDa in length and consists of 107 amino acids which are arranged in two domains of similar folding, giving SLPI a boomerang shape [2, 3]. Each domain is homologous to the whey acidic protein (WAP) four-disulphide core (WFDC) domain [4]. SLPI is constitutively expressed in the majority of mucosal secretions including nasal, bronchial, salivary, tear, cervical, and seminal secretions [5–8]. SLPI is produced and secreted by a number of different cell types including neutrophils and macrophages and by the epithelial cells that line mucosal surfaces. The physiological concentration of SLPI in saliva ranges from 4 to 24 *μ*g/mL [9]. Lung epithelial lining fluid levels of SLPI can be as high as 10 *μ*g/mL, although SLPI concentrations have been found to be higher in upper respiratory airways than in lower airways [1, 9, 10]. The production of SLPI in such defense sensitive locations where protection against pathogen is pivotal reinforces the important role SLPI plays in innate immune defense.

SLPI inhibits a wide variety of proteases including neutrophil elastase (NE), cathepsin G, trypsin, chymotrypsin, chymase, and tryptase [11]. In addition, research suggests that SLPI has antimicrobial effects and is capable of inhibiting both Gram positive (*Staphylococcus aureus*) and Gram negative (*Escherichia coli*) bacterial growth [12, 13] as well as inhibiting HIV viral replication in monocytic cells [14, 15]. In relation to immunomodulation, Jin et al. have shown that SLPI expression in macrophages is induced by lipopolysaccharide (LPS) and suppressed by IFN-*γ* [16]. SLPI inhibited LPS-induced NF-*κ*B activation in macrophages thereby decreasing production of TNF-*α* and IL-8 [17]. Ding et al. showed that SLPI can interfere with uptake of LPS into macrophages [18]. SLPI has been demonstrated to prevent the degradation of key regulatory proteins IRAK, I*κ*B*α*, and I*κ*B*β* in response to LPS and lipoteichoic acid [19–21]. In addition, SLPI exerts a direct effect by entering the nucleus and competing with the p65 subunit of NF-*κ*B for binding to its promoter regions in target genes. Therefore, LPS-induced binding of p65 to the NF-*κ*B consensus-binding sites in the promoter regions of these genes is prevented and production of proinflammatory factors such as TNF-*α* and IL-8 is decreased [17].

To date, there has been very little research in the area of SLPI's ability to inhibit apoptosis. One study has shown that SLPI can inhibit neutrophil apoptosis [22] but the mechanism for this effect is unknown. In this study, we demonstrate that SLPI can inhibit apoptosis in monocytic cells in a way that does not depend on the protease inhibition effect of SLPI.

## 2. Materials and Methods

### 2.1. Materials

The SLPI mutants Lys72SLPI and Gly72SLPI [23] were provided by Amgen (Thousand Oaks, Ca, USA).

### 2.2. Cell Culture

Ethical approval for use of peripheral blood monocytes (PBMs) from whole blood was given by Beaumont Hospital Ethics Committee. Human myelomonocytic U937 cells were purchased from the American Type Culture Collection (Manassas, USA). PBMs and U937s were routinely cultured in RPMI 1640 medium supplemented with 10% heat-inactivated foetal calf serum (Gibco, Life Technologies), 2 mM L-glutamine, and 1% (v/v) penicillin/streptomycin (PAA laboratories GmbH, Austria). Cells for experiments were seeded at 5 × 10^5^/mL and were preincubated with SLPI, oxidised SLPI, or SLPI mutants for 1 hr followed by incubation with TNF-*α* (R&D Systems; 10 ng/mL).

### 2.3. Caspase Activity Assays

Caspase-3 and caspase-7 activity was determined using the fluorogenic substrate Ac-Asp-Glu-Val-Asp-7-amino-4-methylcoumarin (DEVD-AMC; Enzo Life Sciences LTD, Exeter, UK). Briefly, cells were centrifuged at 1000 ×g for 5 minutes at 4°C and lysed in lysis buffer (50 mM Tris pH 7.5, 150 mM NaCl, 5 mM EDTA, and 0.2% Nonidet P-40). The cells were then centrifuged at 17,000 ×g or 10 min at 4°C. The reaction buffer was 10 mM HEPES (pH7.5), 50 mM NaCl, 5 mM MgCl_2_, 2.5 mM DTT, and 1 mM EDTA. Samples were incubated with substrate (50 *μ*M) and fluorescence (substrate turnover) was determined by excitation at 360 nm and emission at 460 nm in a 96-well microplate reader. The rate of substrate hydrolysis was monitored at 37°C and results were expressed as the change (Δ) in relative fluorescence units (ΔRFU) over a 60 min period.

### 2.4. Western Blotting

Whole cell extracts were prepared for Western blotting analysis by lysing cell lines and PBECs in RIPA buffer (50 mm Tris-HCl, pH 7.5, 150 mm NaCl, 1% Nonidet P-40, 0.25% sodium deoxycholate, 1 mm EDTA, and 1% Triton X-100) supplemented with PMSF, aprotinin, and sodium orthovanadate. Total protein concentrations were determined using the BCA method (Pierce BCA Assay, Fisher Scientific UK, Leicestershire). Denatured samples were separated by electrophoresis on 12% SDS-polyacrylamide gels, transferred to nitrocellulose membrane, and probed using rabbit anti-caspase-3, rabbit anti-caspase-7 (Cell Signalling, 1 : 1000), or rabbit anti-GAPDH (Santa Cruz Biotechnology Inc., 1 : 1000). Binding was detected using the appropriate horseradish peroxidase-conjugated secondary antibodies (Fisher Scientific UK), visualized by chemiluminescence (GE Healthcare UK, Buckinghamshire), and analysed using the Syngene G:Box and GeneSnap software (SynGene UK, Cambridge).

### 2.5. Cell Death ELISA

The cell death ELISA kit (Roche) was used to quantify apoptosis levels. The ELISA was carried out according to the manufacturer's instructions.

### 2.6. Oxidation of SLPI

Oxidation of SLPI was carried out as described previously [19].

### 2.7. Statistical Analysis

All data were analysed using GraphPad Prism 5.0 (GraphPad Software Inc., San Diego, CA) and are reported as mean ± SEM. Results are representative of at least *n* = 3 unless otherwise indicated. Means were compared by unpaired *t*-test or one-way analysis of variance (ANOVA) as appropriate. *p* < 0.05 was accepted to indicate statistical significance.

## 3. Results

### 3.1. SLPI Inhibits TNF-*α*-Induced Caspase-3 and Caspase-7 Activity in Monocytic Cells

**Figure 1:**
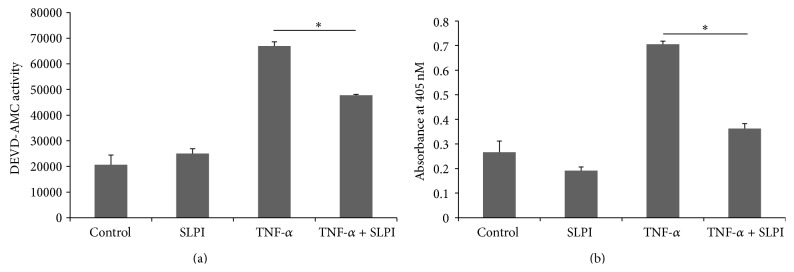
Effect of SLPI on TNF-*α*-induced caspase-3 and caspase-7 activity and cell death. U937 monocytic cells were pretreated for 1 hr with SLPI (10 *μ*g/mL) and stimulated with TNF-*α* (10 ng/mL) for 4 hr. (a) Activity of the effector caspases, caspase-3 and caspase-7, was measured using the fluorogenic substrate DEVD-AMC. Results are representative of *n* = 6. (b) The amount of apoptosis occurring in cells was measured by ELISA. Results are representative of *n* = 6 experiments. *∗* indicates *p* < 0.05.

As effector caspases are responsible for most of the physiological changes that occur in apoptosis, SLPI's effect on effector caspase-3 and caspase-7 activity was initially investigated using the specific substrate DEVD-AMC (Figure 1(a)). U937 monocytes treated with TNF-*α* showed an increase in DEVD-AMC activity compared to control cells, indicating upregulation in caspase-3 and caspase-7 activity in response to TNF-*α* stimulation. In comparison to cells treated with TNF-*α* alone, a significant decrease in caspase-3 and caspase-7 activity was observed in U937 monocytes pretreated with SLPI. This result suggests that SLPI is having an antiapoptotic effect in TNF-*α* treated monocytes.

### 3.2. SLPI Inhibits TNF-*α*-Induced Cell Death in Monocytic Cells

SLPI's effect on TNF-*α*-induced apoptosis was investigated further using a cell death ELISA. The presence of mono- and oligonucleosomes in the cytoplasm of apoptotic cells is due to DNA degradation and occurs several hours before the plasma membrane breakdown. This assay detects the amount of mono- and oligonucleosomes in the cytoplasmic fraction of cell lysates permitting detection of apoptosis. U937 monocytes treated with TNF-*α* showed an increase in the amount of apoptosis as expected which was significantly decreased in the presence of SLPI (Figure 1(b)). This result is consistent with previous findings that SLPI has an antiapoptotic effect on TNF-*α* treated monocytes.

### 3.3. SLPI Inhibits TNF-*α*-Induced Caspase Activation in Peripheral Blood Monocytes

**Figure 2:**
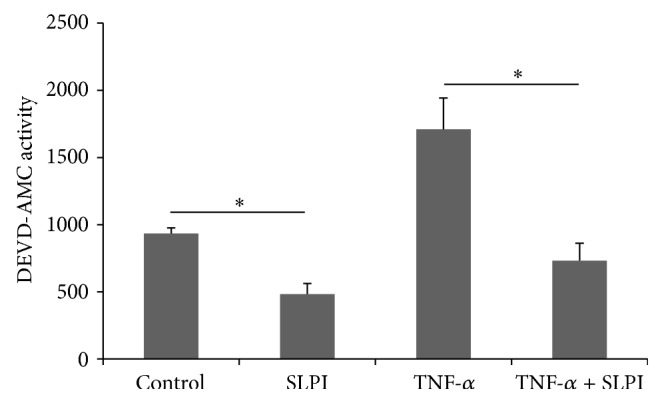
Effect of SLPI on TNF-*α*-induced caspase-3 and caspase-7 activity in peripheral blood monocytes. Peripheral blood monocytes were isolated and pretreated for 1 hr with SLPI (10 *μ*g/mL) and then incubated with TNF-*α* (10 ng/mL) for 4 hr. Activity of the effector caspases, caspase-3 and caspase-7, was measured using the fluorogenic substrate DEVD-AMC. Results are representative of *n* = 6. *∗* indicates *p* < 0.05.

To ensure that the results found in the U937 cell line were physiologically representative of monocytes, peripheral blood monocytes (PBMs) were isolated and treated with TNF-*α* alone and in the presence of SLPI. PBMs treated with SLPI exhibited a significant reduction in basal and TNF-*α*-induced caspase activity (Figure 2). This suggested that results obtained with the U937 cells were physiologically relevant.

### 3.4. The Role of SLPI's Antiprotease Activity in the Inhibition of TNF-*α*-Induced Apoptosis

**Figure 3:**
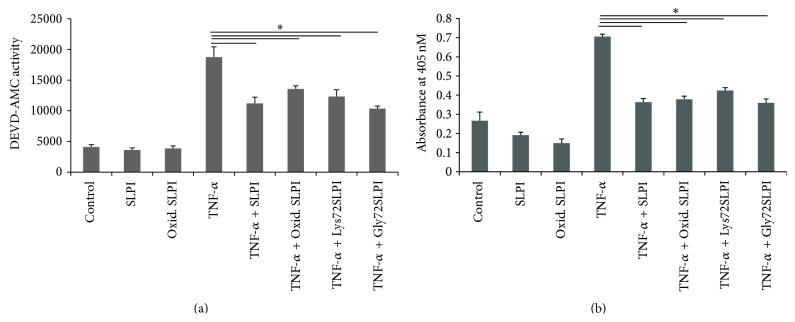
Effect of SLPI and oxidised SLPI on apoptosis in monocytic cells. U937 cells were pretreated with SLPI (10 *μ*g/mL) or oxidised SLPI (10 *μ*g/mL) and stimulated with TNF-*α* (10 ng/mL) for 4 hr. (a) Activity of the effector caspases, caspase-3 and caspase-7, was measured using the fluorogenic substrate DEVD-AMC. (b) The amount of apoptosis occurring in cells was assessed by ELISA (Roche). Results are representative of *n* = 3. *∗* indicates *p* < 0.05.

The effect of antiprotease-deficient variants of SLPI (Lys72SLPI, Gly72SLPI, and oxidised SLPI) [19, 23] on the inhibition of apoptosis was investigated. Lys72SLPI, Gly72SLPI, and oxidized SLPI retained the ability to inhibit TNF-*α*-induced apoptosis as evidenced by caspase-3 and caspase-7 activity assays (Figure 3(a)) and cell death ELISA results (Figure 3(b)). These findings suggest that the antiapoptotic ability of SLPI is independent of its antiprotease activity.

### 3.5. Investigation into SLPI's Mechanism of Apoptosis Inhibition

**Figure 4:**
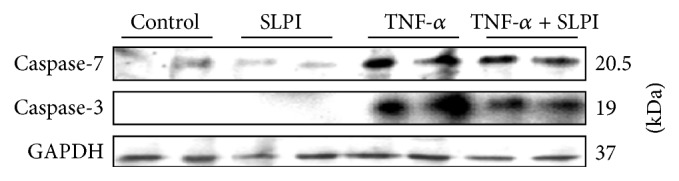
Effect of SLPI on active caspase-3 and caspase-7 protein levels. U937 cells were pretreated with SLPI (10 *μ*g/mL) and stimulated with TNF-*α* (10 ng/mL) for 4 hr. RIPA extracts were electrophoresed on 10–15% SDS-PAGE gels and analysed by Western blotting using anti-caspase-3 and anti-caspase-7 antibodies. The blots were tripped and reprobed for GAPDH to control for protein loading. Results are representative of *n* = 3.

**Figure 5:**
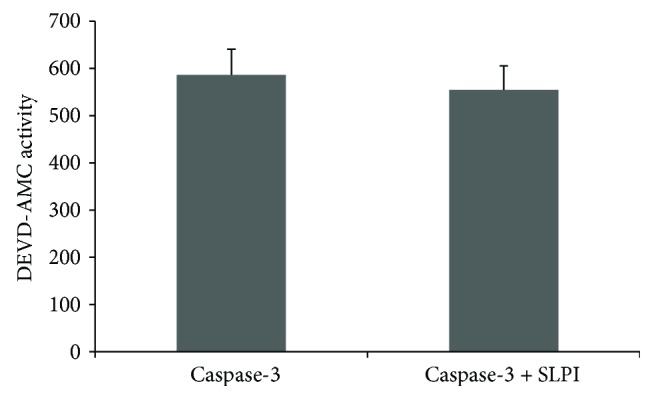
Effect of SLPI on recombinant caspase-3 activity. SLPI (100 ng) was pretreated for 1 hr with recombinant human caspase-3 (10 ng). Caspase-3 activity was measured using the fluorogenic substrate DEVD-AMC. Results are representative of *n* = 5.

U937 cell lysates were prepared from cells treated with TNF-*α* alone and in the presence of SLPI and levels of active caspase -3 and caspase-7 investigated by Western blotting. Despite the fact that there was a decrease in caspase-3 and caspase-7 activity (Figure 1(a)), active forms of both caspases were found in cells treated with TNF-*α* in the presence of SLPI as shown in Figure 4. However, the levels of active caspase-3 appear reduced in cells treated with TNF-*α* and SLPI compared to cells stimulated with TNF-*α* alone. To address the possibility that SLPI may directly inhibit caspase-3 activity, recombinant caspase-3 was pretreated with a molar excess of SLPI prior to the addition of DEVD-AMC. As illustrated in Figure 5, no inhibition of active caspase-3 was detected.

## 4. Discussion

SLPI has been shown to inhibit LPS-induced NF-*κ*B activation by displacing or inhibiting NF-*κ*B binding to its promoter regions in target genes [17]. As well as inducing a host of proinflammatory cytokines, NF-*κ*B activation also leads to the induction of prosurvival factors, which combine to inhibit apoptosis. Therefore, the effect of SLPI on TNF-*α*-induced apoptosis was investigated to see if SLPI was capable of inhibiting NF-*κ*B antiapoptotic effects. We induced apoptosis using TNF-*α* which has previously been shown to have proapoptotic effects in monocytic cells [24]. Surprisingly, SLPI was shown to downregulate caspase-3 and caspase-7 activity and inhibit TNF-*α*-induced apoptosis in monocytes. This effect was further investigated using the cell death ELISA which confirmed that SLPI inhibited TNF-*α*-induced cell death.

Both NE and cathepsin G have previously been shown to play a role in apoptosis [25, 26]. Therefore, we hypothesised that, as a serine protease inhibitor, SLPI inhibits apoptosis via NE or cathepsin G inhibition. To test this, we evaluated antiprotease deficient variants of SLPI (Lys72SLPI, Gly72SLPI, and oxidised SLPI) [19, 23]. The antiprotease inactive forms of SLPI retained the ability of inhibiting TNF-*α*-induced apoptosis suggesting that SLPI's antiapoptotic effects were not mediated by its antiprotease activity. In relation to SLPI's anti-inflammatory activity, the literature is conflicting regarding the role that SLPI's antiprotease function plays in mediating these effects [27, 28].

In a bid to understand how SLPI may mediate its antiapoptotic activity, the effect of SLPI on other components of the apoptotic pathway was then investigated. Similar to SLPI, PR39 is a small, highly cationic, and antimicrobial peptide which can also enter cells and inhibit apoptosis. PR39 achieves this through the upregulation of the antiapoptotic protein IAP-2 [29]. IAP-2 and other IAP family members inhibit caspase-3 and caspase-7 activity. We therefore hypothesised that SLPI could act in a similar manner to PR39 by upregulating IAP family members which could inhibit caspase-3 and caspase-7 directly. We looked at the effect of SLPI on IAP-1, IAP-2, survivin, and XIAP protein levels but found that SLPI did not upregulate any of these factors (data not shown). In addition, SLPI was unable to directly inhibit recombinant caspase-3 activity. However, as the levels of active caspase-3 appear lower in cells treated with TNF-*α* and SLPI compared to cells stimulated with TNF-*α* alone, it is possible that at least part of SLPI's antiapoptotic mechanism may involve inhibition of the generation of active caspase-3 from its inactive zymogen perhaps via inhibition of upstream initiator caspases such as caspase-8. Further work is required to elucidate the exact mechanism by which SLPI exerts its antiapoptotic effects in response to TNF-*α*. Previous work has reported the ability of SLPI to limit TNF-*α*-induced hydrogen peroxide production by human neutrophils [27, 30]; therefore, it is also possible that SLPI may be able to interfere with more upstream TNF-*α* signalling intermediates.

From a physiological standpoint, SLPI's antiapoptotic effect in monocytes may be important for optimising the removal of dying cells and bacteria as part of the inflammation resolution process and the fight against infection. Conditions such as emphysema and cystic fibrosis, characterised where SLPI levels are altered by degradation [10, 31] in the lung, are dominated by a chronic inflammatory response, excessive apoptosis, and infection. Therefore, SLPI's role in controlling this process on the respiratory tract may be an important homeostatic mechanism in maintaining sufficient monocyte numbers for infection and inflammation resolution. More work is needed to determine the mechanism by which SLPI alters apoptotic pathways.
